# Romantic Love and Behavioral Activation System Sensitivity to a Loved One

**DOI:** 10.3390/bs13110921

**Published:** 2023-11-10

**Authors:** Adam Bode, Phillip S. Kavanagh

**Affiliations:** 1 School of Archaeology and Anthropology, ANU College of Arts and Social Sciences, The Australian National University, Canberra, ACT 2601, Australia; 2 Discipline of Psychology, Faculty of Health, University of Canberra, Bruce, ACT 2617, Australia; phil.kavanagh@canberra.edu.au; 3 Justice and Society, University of South Australia, Magill, SA 5072, Australia

**Keywords:** BAS-SLO Scale, behavioral activation system, CFA, evolution, romantic love, Romantic Love Survey 2022

## Abstract

Research investigating the mechanisms that contribute to romantic love is in its infancy. The behavioral activation system is one biopsychological system that has been demonstrated to play a role in several motivational outcomes. This study was the first to investigate romantic love and the behavioral activation system. In study 1, the Behavioral Activation System—Sensitivity to a Loved One (BAS-SLO) Scale was validated in a sample of 1556 partnered young adults experiencing romantic love. In study 2, hierarchical linear regression was used to identify BAS-SLO Scale associations with the intensity of romantic love in a subsample of 812 partnered young adults experiencing romantic love for two years or less. The BAS-SLO Scale explained 8.89% of the variance in the intensity of romantic love. Subject to further validation and testing, the BAS-SLO Scale may be useful in future neuroimaging and psychological studies. The findings are considered in terms of the mechanisms and evolutionary history of romantic love.

## 1. Introduction

Research investigating the mechanisms that contribute to romantic love is in its infancy. The behavioral activation system (BAS) is one biopsychological system that has been demonstrated to play a role in several motivational outcomes. To our knowledge, no studies have investigated the role the BAS may play in romantic love. Using a biological conceptualization of romantic love, we develop a means of assessing BAS sensitivity to a loved one and assess its association with the intensity of romantic love. The result is the formulation of a new means of assessing one biopsychological system that may contribute to the expression of romantic love.

### 1.1. Romantic Love

The topic of love in romantic relationships is riddled with definitional inconsistency and ambiguity. Sociological [1,2], anthropological [3], psychological [4,5], and biological [6] conceptions of love in romantic relationships all have their own terminology and formulations. While in many such disciplines, it is common to refer to all types of love within romantic relationships as “romantic love,” the biopsychological focus of this article leads us to choose a different approach. In the discipline of biology, “romantic love” tends to refer to the period of intense feelings that often accompanies the early stages of romantic relationships [6,7]. As such, we use the term “romantic love” to refer to a motivational state associated with a range of reproductive functions, including mate choice, courtship, sex, and pair bonding [6] (p. 21). It is the basis of long-term romantic relationships and family formation throughout much of the world. It is associated with a range of cognitive, emotional, and behavioral activities in both sexes. It is sometimes referred to as “passionate love” in certain areas of psychology [8]. The expression of romantic love is partly socially or culturally influenced, and differences in its presentation are found across cultures (e.g., [1,2,3,9,10,11,12]).

Cognitive activity of romantic love includes intrusive thinking or preoccupation with the partner, idealization of the other in the relationship, and desire to know the other and to be known. Emotional activity includes attraction to the other, especially sexual attraction, negative feelings when things go awry, longing for reciprocity, desire for complete union, and physiological arousal. Behavioral activity includes actions toward determining the other’s feelings, studying the other person, service to the other, and maintaining physical closeness.

Romantic love often happens at the early stages of a romantic relationship (referred to as early-stage romantic love) and usually lasts months or years (see [13,14]) but can sometimes last many years or decades (referred to as long-term romantic love) [15,16,17]. The psychological characteristics of both types of romantic love are similar, except that long-term romantic love is not characterized by intrusive thinking or preoccupation with the partner [15,16]. The neural mechanisms that cause each type of romantic love are similar but are not identical.

Romantic love is most strongly associated with neural activity in systems associated with reward and motivation (e.g., ventral tegmental area, nucleus accumbens, amygdala, and medial prefrontal cortex), emotions (e.g., amygdala, anterior cingulate cortex, and the insula), sexual desire and arousal (e.g., caudate, insula, putamen, and anterior cingulate cortex), and social cognition (e.g., amygdala, insula, and medial prefrontal cortex), as well as higher-order cortical brain areas that are involved in attention, memory, mental associations, and self-representation [6,18]. Functional connectivity is increased in people experiencing early-stage romantic love within the reward, motivation, and emotion regulation network (dorsal anterior cingulate cortex, insula, caudate, amygdala, and nucleus accumbens) as well as the social cognition network (temporo-parietal junction, posterior cingulate cortex, medial prefrontal cortex, inferior parietal, precuneus, and temporal lobe [19]. Early-stage romantic love is also associated with lower network segregation and altered connectivity degree [20] and with the endocrinal activity of sex hormones, serotonin, dopamine, cortisol, oxytocin, and nerve growth factor [6]. To our knowledge, no research has investigated the endocrinological correlates of long-term romantic love.

### 1.2. The Behavioral Activation System (BAS)

One biological mechanism that is thought to play a role in the promotion of behavior is the BAS. This system is believed to be associated with dopaminergic reward and motivation circuitry [21,22,23,24]. The BAS works as a system that involves both inputs and outputs. Inputs are stimuli that serve as cues for goal-directed behavior. They include life events involving goal salience or goal attainment. Behavioral activation system outputs include motor activity, energy, confidence, interest, pleasure in rewards, and, potentially, sociability and exploration. The general outputs of the BAS have been compared with symptoms of mania, including initiation to locomotor activity, activity and exploration, and anger (see [25]).

### 1.3. The BAS and Romantic Love

People experiencing romantic love display a range of cognitions, emotions, and behaviors suggestive of heightened BAS activity. These include increased reward valuation, willingness to expend effort to gain reward, heightened initial hedonic response to success in the form of learning deficits, and lack of satiety in response to success (see [25]).

People experiencing romantic love demonstrate an increased reward valuation of the loved one. The loved one takes on a “special meaning” [26] (p. 32). The perception of the loved one changes, and idealization ensues, as does the belief that the loved one is the “perfect romantic partner” [10] (p. 391) for them and that their loved one satisfies their preferred standards of physical attractiveness [27] (p. 395). The loved one becomes the most important person in their life.

People experiencing romantic love appear to demonstrate a willingness to expend effort to gain reward. Romantic lovers often engage in courtship (see [6] for a review of the costs and benefits of courtship among people experiencing romantic love), which involves a series of signals and behaviors that serve as a means of assessing potential partner quality and willingness to invest in a relationship [28,29]. People experiencing romantic love are also willing to reorder daily priorities, make themselves available to their loved one, and take steps to make themselves desirable to their loved one by changing their “clothing, mannerisms, habits, or values” [26] (p. 33).

Some people experiencing romantic love may demonstrate some aspects of heightened initial hedonic response to success in the form of learning deficits. The most cogent example of this is the instances of obsessive pursuit (usually committed by men), which occur in the absence of rewarding interaction from the loved one. Men, in particular, but not exclusively, have a tendency to misinterpret politeness or friendliness for sexual interest from potential sexual partners (see [30] for review). Such a false positive bias is potentially present in people experiencing romantic love and can result in repeated attempts by an individual to court a loved one despite there being obvious indications that such efforts will be fruitless. That both females and males can be subject to ineffective courting demonstrates the potent motivational effect romantic love can have on both sexes. This is one BAS sensitivity component that warrants further investigation in people experiencing romantic love.

People experiencing romantic love demonstrate a lack of satiety in response to success. For example, even when an individual in love feels emotionally close to their loved one, there can be a desire to be even closer. A sense of avolition and uncontrollability is a feature of romantic love [26] (p. 33). This is evidenced by an individual reordering their daily activities to spend increasingly long periods with their loved one and, in the modern environment, the obsessive monitoring of social media pages of the loved one. More generally, people experiencing romantic love experience prolonged affect, confidence, and increased energy over prolonged periods, as is indicated by the hypomanic symptoms found to be present in adolescents experiencing romantic love reported by Brand and colleagues [31].

### 1.4. Salience of Loved One-Related Stimuli and the BAS

There is evidence that when an individual is in love, the loved one takes on a special meaning [26]. This can be considered in terms of loved one-related stimuli having increased salience, probably as a result of oxytocin activity in one or more motivation pathways [32] (see also [33]). This has been demonstrated empirically in terms of memory and attention [34], as well as the heightened BAS sensitivity characteristics of romantic love detailed above. Because the BAS is situated within a motivational system, we believe that this salience of the loved one and loved one-related stimuli means the BAS probably responds in a particularly sensitive manner to loved one-related stimuli.

This heightened salience of loved one-related stimuli among individuals experiencing romantic love suggests that BAS sensitivity, somewhat analogous to anxiety (see [35]), may exist in a trait and state form. General BAS sensitivity may be relatively stable and influence behavior over the life course in a consistent manner. This is a type of trait BAS sensitivity. There are also periods when the BAS may become particularly sensitive, such as during a manic episode (see [25]), or in relation to a particular person, such as in circumstances of romantic love. This is a type of state of BAS sensitivity. The foci of the current studies are this state of BAS sensitivity that is characteristic of romantic love.

### 1.5. The BAS Scale

One common measure of BAS sensitivity is the BAS Scale [36], which includes three subscales: reward responsiveness, drive, and fun-seeking. The BAS Scale was originally validated by Carver and White (1994) in conjunction with items assessing the behavioral inhibition system (BIS) using exploratory factor analysis, Cronbach’s alpha (reliability), and correlation scores with other related measures (convergent validity). The analysis found four factors explained the BIS/BAS Scale: (i) BIS, (ii) BAS reward responsiveness, (iii) BAS drive, and (vi) BAS fun-seeking. More recently, efforts using confirmatory factor analysis have been undertaken to confirm the reliability of the BAS Scale (e.g., [37,38,39,40,41,42,43,44]) and a single factor (two-factor BIS/BAS Scale) has been suggested (e.g., [38,39,43]) with some degree of support [38,43].

The reward-responsiveness subscale assesses the tendency to respond to rewards with energy and enthusiasm, the drive subscale assesses motivation to pursue goals, and the fun-seeking subscale assesses the tendency to pursue positive experiences without regard to potential threats or costs [36] (see [25] for a summary of findings in relation to BAS Scale subscales and bipolar disorder). It seems feasible that all three subscales could contribute to aspects of romantic love, as the BAS responds to loved one-related stimuli.

### 1.6. The Current Studies

This is the first attempt to investigate the Behavioral Activation System and romantic love. As a result, we undertake preliminary work to shed light on the relationships between these two constructs. We amended the BAS Scale to assess *BAS Sensitivity to a Loved One* (BAS-SLO; described below). In Study 1, we validate the BAS-SLO Scale. This was a necessary step in developing an initial understanding of the relationship between the behavioral activation system and romantic love. We used confirmatory factor analysis to assess the suitability of three factor structures: (i) a one-factor model; (ii) a three-factor, 13-item structure; and (iii) a three-factor, 12-item structure. We determined that a three-factor, 12-item structure possessed the best goodness of fit. We calculated Cronbach’s alphas to test internal reliability and correlated subscales with a related measure to assess convergent validity for this structure. In Study 2, we tested the hypothesis that the BAS-SLO Scale will be positively associated with the intensity of romantic love. Findings are considered within an evolutionary framework, which helps elucidate the mechanisms and evolutionary history of romantic love.

## 2. Study 1: Validating the BAS-SLO Scale

### 2.1. Materials and Methods

#### 2.1.1. Participants

Participants were 1556 English-speaking young adults who self-identified as being in love taken from the Romantic Love Survey 2022 [45]. Appendix A presents the characteristics of participants used in Study 1 and the country of residence of participants. We use the majority of the ideas for sample characteristics reporting from Bode and Kowal [7].

#### 2.1.2. Measures

The Behavioral Activation System Sensitivity to a Loved One (BAS-SLO) Scale was created by amending each item of the BAS Scale to relate to an individual’s loved one or relationship with their loved one. Participants were asked, “Indicate how much the following applies to you”. Responses were scored on a four-point scale (1 = very true for me; 4 = very false for me). Scores for each item are reverse coded, and subscale scores are summed. Table 1 presents the original BAS Scale items and the BAS-SLO Scale items for each subscale.

We also used the Passionate Love Scale—30 (PLS-30) to assess the convergent validity of the BAS-SLO scale. The PLS-30 is a 30-item measure of the cognitive, emotional, and behavioral characteristics of romantic love. Each item records scores by assessing agreement with statements on a nine-point Likert scale (1 = not at all true; 9 = definitely true). It is the most commonly used measure of romantic love in biological studies of romantic love [7]. Cronbach’s alpha for the PLS-30 in this sample was 0.944.

#### 2.1.3. Procedure

We conducted a confirmatory factor analysis (CFA) on the 13 items of the BAS-SLO Scale using techniques/suggestions from a guidance paper [46]) and predicted a three-factor solution in line with the original BAS Scale factor structure [36]. A CFA using a one-factor solution was also conducted, as there is some literature suggesting that the BAS can be explained by a single factor [38,39,43]. At the suggestion of one reviewer, following an initial round of peer review, we then conducted another three-factor CFA of 11 items from the proposed BAS-SLO (removing two poorly loaded items; reward responsiveness item 5 and fun-seeking item 3).

A weighted least square mean and variance adjusted (WLSMV) method of confirmatory factor analysis was used as the data were ordinal [47]. The comparative fit index (CFI), standardized root-mean-square error of approximation (RMSEA), and standardized root-mean-square residual (SRMR) were used to assess the appropriateness of all three models in accordance with common practice (see [46]).

The following criteria, based on work by Hu and Bentler [48] and the model CFA example by Knetka and Runyon [46], were used to assess the adequacy of the model: CFI > 0.95 (although 0.90 is required to ensure mis-specified models are not deemed acceptable), RMSEA < 0.06, and SRMR < 0.08. Internal reliability was assessed by calculating Cronbach’s alpha for each BAS-SLO subscale. Values of >0.70 were considered acceptable (see [49]). We assessed convergent validity by correlating BAS-SLO Scale subscales (i.e., reward responsiveness, drive, and fun-seeking) with the PLS-30 and the amended HCL-32. Factor loadings, covariances, and goodness of fit indices were calculated using the Lavaan package for R version 4.2.2 in R Studio. The CFA diagram was created in AMOS version 26. Convergent validity analyses were conducted using SPSS version 27.

### 2.2. Results

No items from the BAS-SLO were missing data. Two cases were missing data for the PLS-30. These two cases were not included in the correlation analysis. Table 2 presents the means, standard deviations, skewness statistics, and kurtosis statistics for the 13 items of the BAS-SLO. Most of the data were moderately skewed, but this was deemed acceptable as the robust maximum likelihood method has been shown to be robust against violations of normality (see [47]).

#### 2.2.1. Three-Factor, 13-Item Model

Results from the three-factor 13-item CFA indicated that, in our sample, the model had adequate but not good psychometric properties (see Appendix B for a summary table of goodness of fit statistics for all models). CFI was 0.944, indicating an acceptable (but not quite good) fit. RMSEA was 0.055, indicating good fit. SRMR was 0.041, indicating good fit. Factor loadings ranged from 0.44 to 0.80, with the majority above 0.60 (see Appendix C), suggesting that the factors explained most of the items reasonably (but not very) well. Factors correlated with each other from 0.40 (drive and fun-seeking) to 0.66 (reward responsiveness and fun-seeking), suggesting the discriminate validity was acceptable. Two items (R5 and F3) loaded poorly onto the reward responsiveness and fun-seeking factors (0.44 and 0.52), respectively. Appendix C presents the results of the three-factor, 13-item CFA.

#### 2.2.2. One-Factor, 13-Item Model

Results from the one-factor, 13-item CFA indicated that, in our sample, the model had very poor psychometric properties. CFI was 0.709, indicating very poor fit. RMSEA was 0.121, indicating very poor fit. SRMR was 0.086, indicating poor fit. Because this model had very poor fit, we do not report further on the results.

#### 2.2.3. Three-Factor, 11-Item Model

**Figure 1:**
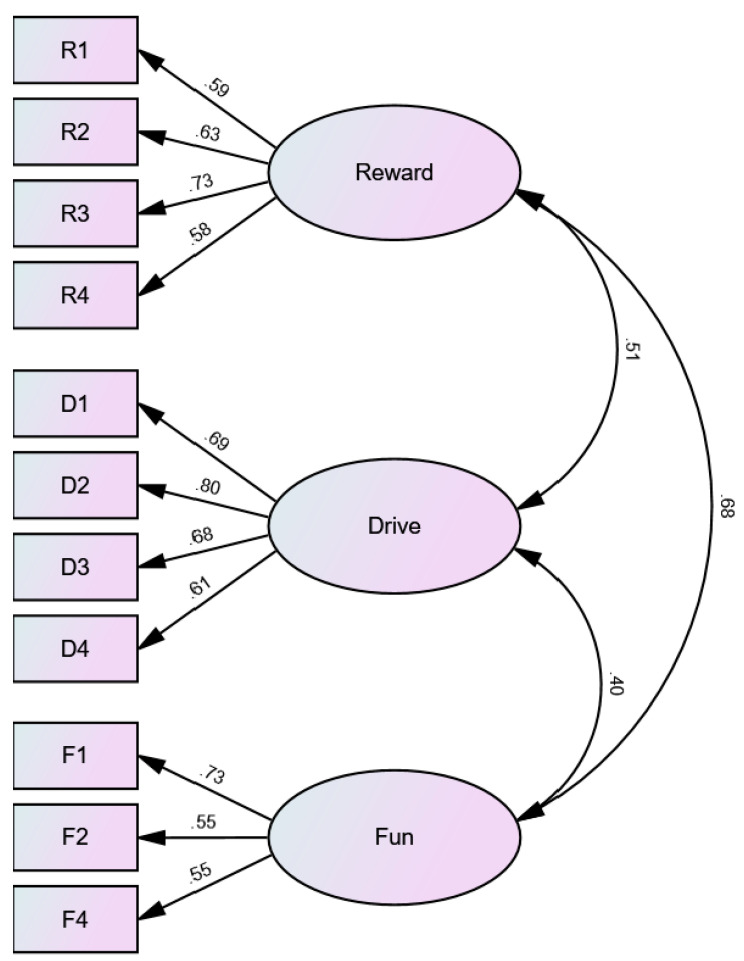
Results of the three-factor, 11-item CFA model for the BAS-SLO Scale. Note: Survey items (for description, see Table 3) are represented by rectangles, and latent factors are represented by ovals. Reward = reward responsiveness; Drive = drive; Fun = fun-seeking. The numbers above the one-directional arrows connecting factors to items represent standardized factor loadings. The numbers to the right of the bi-directional arrows connecting factors represent correlations between the factors.

Because R5 and F3 loaded substantially lower than all the other items in the three-factor, 13-item CFA, we removed these items and ran another three-factor CFA, this time with 11 items. Results indicated that, in our sample, the three-factor, 11-item model had good psychometric properties, but loadings were not generally improved from the three-factor, 13-item CFA. CFI was 0.966, indicating good fit. RMSEA was 0.048, indicating good fit. SRMR was 0.037, indicating good fit. Factor loadings ranged from 0.55 to 0.80, with the majority above 0.60 (see Figure 1), suggesting that the factors explained most of the items reasonably (but not very) well. Factors correlated with each other from 0.40 (drive and fun-seeking) to 0.68 (reward responsiveness and fun-seeking), suggesting the discriminate validity was acceptable. Figure 1 presents the results of the three-factor, 11-item CFA.

Cronbach’s alpha for the three-factor, 11-item BAS-SLO Scale was 0.725 for reward responsiveness, indicating acceptable internal reliability; 0.786 for drive, indicating acceptable internal reliability; and 0.629 for fun-seeking, indicating marginally questionable internal reliability. Cronbach’s alphas for all subscales aligned closely with those of the original BAS subscales (reward responsiveness = 0.73, drive = 0.76, fun-seeking = 0.66; [36]) and with subsequent studies (e.g., [37,39]).

Convergent validity was assessed by correlating each of the BAS-SLO Scale subscales with the PLS-30. We anticipated that each BAS-SLO Scale subscale would correlate highly with the PLS-30. Table 3 presents the correlations between the BAS-SLO Scale subscales and the PLS-30. PLS-30 had a large association with reward responsiveness and a medium association with drive and fun-seeking. This suggests good convergent validity.

### 2.3. Discussion

Study 1 reported three CFAs of the BAS-SLO Scale. A three-factor model for the BAS-SLO Scale with 11 items that aligned with the three factors of the original BAS Scale (reward responsiveness, drive, and fun-seeking) was deemed to be an appropriate model by CFA, as well as the reliability and convergent validity analyses. This is especially the case when considered in light of the psychometric properties of the original BAS Scale and subsequent studies indicating a three-factor model of the BAS Scale utilizing confirmatory factor analysis (e.g., [42,43,44]). Indices of fit generally supported the notion of an acceptable model with good fit. Factor loadings were lower than would be ideal, suggesting the factors did not explain the data well. Correlations among the factors suggest the discriminate validity of the BAS-SLO is moderately low. Internal reliability of the subscales ranged from marginal to acceptable. This is in line with alphas for these three subscales in previous studies [42,43,44]. Correlations between subscales of the BAS-SLO Scale and the PLS-30 were roughly as expected, suggesting good convergent validity. In sum, we think the BAS-SLO is a measure that could be used in studies investigating BAS sensitivity to a loved one and romantic love, as well as a range of other related phenomena. Appendix D presents the final items of the proposed BAS-SLO Scale.

## 3. Study 2: The BAS and Romantic Love

### 3.1. Materials and Methods

#### 3.1.1. Participants

Participants were a subsample of study 1 participants, 812 English-speaking young adults who self-identified as being in love from the Romantic Love Survey 2022 [45]. Participants who had been in love for 23 months or less and scored above 130 on the PLS were included in the analysis. Two years is a likely period of time in which individuals experience early-stage romantic love rather than long-term romantic love (see [6]). Two cases were missing one data point, and these cases were removed. One intersex participant was removed. Appendix E presents the characteristics of participants used in Study 2 and the country of residence of the participants. We use the majority of the ideas for sample characteristics reporting from Bode and Kowal [7].

#### 3.1.2. Measures

Behavioral Activation System sensitivity to a loved one was measured using the three subscales of the 11-item BAS-SLO Scale validated in Study 1. Intensity of romantic love was measured using the Passionate Love Scale (PLS-30; [10]; described in Study 1). *Sex* was measured using a simple question asking, “What is your biological sex?” Data were coded as 1 (female) or 2 (male). Some studies have suggested that females experience romantic love marginally more intensely than males [50,51]. Love in romantic relationships has been thought to follow a specific trajectory of intensity related to intimacy, passion, and commitment [52]. As such, the length of time an individual has been in love may be associated with the waxing or waning intensity of romantic love. *Months in love* was assessed by asking participants how long they had been in love with their loved one. Obsessive thinking is definitive of early-stage romantic love (see [10,53,54]) and one proposed biological component of romantic love [33]. It therefore follows that it may have a direct influence on the intensity of romantic love. Percent of time thinking about a loved one (*obsessive thinking*) was measured by asking participants, “What percentage of your waking hours do you spend thinking about the person you love?” Responses were on a scale from 0% to 100%. *Commitment* was measured by using five items from the TLS-15 commitment subscale [55] but with a nine-point scoring approach. Each item records scores by assessing agreement with statements ranging from 1 (not at all) to 9 (extremely). Romantic love is believed to serve as a commitment device [56,57] and, therefore, may have a direct association with the intensity of romantic love.

#### 3.1.3. Procedure

To test the hypothesis that BAS sensitivity to a loved one would predict romantic love, we undertook a hierarchical linear regression whereby the BAS-SLO Scale predicted PLS-30. Step one included controls. Step 2 included controls and each of the three BAS-SLO Scale subscales.

### 3.2. Results

Table 4 reports the correlations among all variables used in Study 2 analyses and their descriptive statistics.

Our hypothesis predicted that the BAS-SLO Scale would be positively associated with the intensity of romantic love. To test this hypothesis, we undertook a hierarchical linear regression whereby the BAS-SLO Scale predicted PLS-30 scores after controlling for sex, months in love, obsessive thinking, and commitment. All assumptions for linear regression were met. The hierarchical linear regression predicting the intensity of romantic love revealed that, at Step 1, control variables contributed significantly to the regression model, with *F*(6, 805) = 166.987 and *p* < 0.001, and accounted for 45.02% of the variance in intensity of romantic love. Adding the BAS-SLO Scale to the regression model (Step 2) explained an additional 8.89% of the variation in the intensity of romantic love, and this change in adjusted *R*^2^ was significant; *F*(3, 802) = 136.519 and *p* < 0.001. Each individual BAS-SLO Scale subscale contributed significantly to the model (reward responsiveness, *p* < 0.001; drive, *p* < 0.001; and fun-seeking, *p* = 0.017). Table 5 presents the regression statistics for this analysis.

### 3.3. Discussion

Study 2 used the BAS-SLO Scale to examine the associations between the BAS sensitivity to a loved one and the intensity of romantic love in young adults experiencing romantic love for less than two years. We hypothesized that the BAS sensitivity to a loved one would be positively associated with the intensity of romantic love. Our hypothesis was confirmed. The BAS-SLO Scale explained 8.89% of the variance in the intensity of romantic love (measured by the PLS-30), confirming our hypothesis. This amounts to a medium effect [58] of BAS sensitivity to a loved one on the intensity of romantic love. All three subscales contributed significantly to the model, and this suggests that the BAS plays a role in romantic love. That all three subscales contributed to the model raises the question as to whether each subscale contributes to specific components that characterize the intensity of romantic love in the PLS-30.

The findings of Study 2 are important because they demonstrate that the BAS-SLO Scale may be useful in investigating romantic love and provide the first evidence that the BAS plays a role in romantic love. The findings suggest that future studies may be able to identify the unique components of romantic love caused by the BAS and its state of sensitivity to a loved one. Future studies could use the BAS-SLO Scale to predict individual features of the intensity of romantic love. The use of the BAS-SLO Scale could also potentially be extended to investigate aspects of established pair bonds and relationships characterized by pair bond maintenance and not characterized by the presence of pair bond formation and romantic love (see [33]). Further, the BAS-SLO Scale’s use could be combined with fMRI analyses to identify the neurobiological components of the BAS and their contribution to romantic love.

This study is not without limitations, however. The sample is constituted entirely of young adults in the first two years of romantic love. As a result, the sample is neither representative of the entirety of the human population who experiences romantic love nor the entire spectrum of romantic love (see [7] for issues of generalizability). Further, the analysis was undertaken on a subsample of that used to validate the Scale in Study 1. This limits the implications of the findings, given that the Scale may possess different properties in a different sample. Nonetheless, the study has demonstrated the potential usefulness of the BAS-SLO Scale and provided the first evidence that the BAS plays a role in romantic love.

## 4. General Discussion

This article presents the first direct evidence of the relationships between BAS sensitivity and romantic love. Study 1 demonstrated that it is possible to measure BAS sensitivity to a loved one. Study 2 demonstrated that this means of measurement can be useful in empirical studies investigating the relationship between the BAS and romantic love. Combined, these two studies suggest that BAS sensitivity to a loved one is a real phenomenon and that the state of romantic love is probably associated with BAS sensitivity to a loved one. This has implications for understanding the mechanisms and evolutionary history of romantic love (see [59]).

The reason the BAS can be particularly sensitive to a loved one may relate to the concept of salience. Froemke and Young [32] have suggested that oxytocin acts on motivation pathways to increase the salience of specific social stimuli. In humans, this may take place in the ventral tegmental area (VTA). The VTA is consistently implicated in fMRI studies of people experiencing romantic love (see [6,60,61]). Although void of oxytocin receptors, the human VTA has been identified as the area in which oxytocin attaches salience to socially rewarding cues [62]. This increased salience probably results in further up-regulation of dopamine pathways, presumably including those that characterize the activity of the BAS. This supports Bode’s [33] contention that the bonding attraction system in romantic love is characterized by both oxytocin and dopamine activity, among other factors.

Several studies have shed light on the neural structures associated with BAS sensitivity (assessed with the BAS Scale) in normal samples. BAS sensitivity has been associated with activity in the VTA–nucleus accumbens pathway and the orbitofrontal cortex [21], and BAS reward responsiveness has been associated with lateral prefrontal cortex, anterior cingulate cortex, and ventral striatum [22] in healthy samples. Interestingly, BAS drive has been associated with less activity in the putamen, caudate, and thalamus, and BAS reward responsiveness has been associated with increased activity in the left precentral gyrus in response to different intensities of infant cries among mothers [23]. Variation in regional gray matter volume in the ventromedial prefrontal cortex and inferior parietal lobule has also been associated with BAS Scale scores [24]. There is also evidence that reward network glutamate levels contribute to individual differences in BAS reward responsiveness [63]. These structures generally overlap with those found in romantic love (see [6]).

Knowledge about the neural structures associated with BAS sensitivity, their overlap with the structures associated with romantic love, and now, a means of measuring BAS sensitivity to a loved one provide the means of measuring specific bio-psychological mechanisms that likely contribute to romantic love. Functional magnetic resonance imaging studies can begin to isolate the specific contribution of the BAS to the intensity of romantic love or specific features of romantic love. The implications of the studies reported in this article extend beyond a better understanding of the mechanisms of romantic love. They also provide insights into the evolutionary history of romantic love.

The findings support the notion that romantic love evolved by using pre-existing neural mechanisms (see [6]). The BAS is evolutionarily old, and romantic love made use of this system in a novel way. Instead of increasing general sensitivity, it generates a salience of a particular social stimulus (the loved one), which in turn increases sensitivity to the loved one. This increased salience is possibly the same mechanism that results in increased sensitivity among a plethora of other mechanisms. For example, evidence that lovers have an attentional and memory bias towards loved one-related stimuli [34] suggests that the stimuli possess greater importance or value than other stimuli. This is not the result of state changes to the attentional or memory system but rather the result of increased salience of loved one-related stimuli. This is a simple and elegant way of recruiting cognitive, emotional, and behavioral efforts in response to stimuli that have been identified at the input to be of great importance. This salience presumably required an internal schema of the loved one, an assessment of stimuli to identify their concordance, and then the application of increased value or weight to those stimuli. The concepts of salience and sensitivity are fundamental to a better understanding of the mechanisms of romantic love.

This process of increasing salience is probably at the core and very beginning of the evolution of romantic love and may be associated with the left VTA [61]. Mutation permitted the increased valuation of particular social stimuli, and that was possibly the first step in its evolutionary history. The particular features of romantic love, and perhaps some of its functions, such as pair bonding, may have evolved long after this initial step. Courtship attraction, which is also associated with an increased salience of social stimuli (see [33]), and sexual desire probably become intertwined with romantic love over the following generations. This is in line with previous suggestions that a precursor to contemporary romantic love emerged prior to the evolution of pair bonds [33,64].

The findings of these two studies also highlight the likelihood that BAS sensitivity exists in both a trait and state manner. The traditional BAS Scale assesses dispositional trait sensitivity, whereas the BAS-SLO Scale assesses what can be considered a type of state sensitivity. Parallels with anxiety may help to guide future researchers when elucidating these distinct but related phenomena. To better understand the similarities and differences between trait and state BAS sensitivity, it will be necessary to identify the role of trait sensitivity in romantic love.

## 5. Conclusions

This article reported two studies related to the behavioral activation system and romantic love. In Study 1, the BAS-SLO Scale was validated in a sample of 1556 partnered young adults experiencing romantic love. The validation determined that the characteristics of the BAS-SLO Scale were sufficient to justify its use in future psychological and imaging studies. In study 2, hierarchical linear regression was used to identify BAS-SLO Scale associations with the intensity of romantic love in a subsample of 812 partnered young adults experiencing romantic love for two years or less. The BAS-SLO Scale explained 8.89% of the variance in the intensity of romantic love. The findings shed light on one of the biopsychological mechanisms that contribute to romantic love and provide insights into the specific functions of regions associated with romantic love from fMRI studies. The BAS-SLO Scale should be used in future psychological and imaging studies.

## Figures and Tables

**Table 1:** Original BAS Scale items and the equivalent BAS-SLO Scale items.

Item	BAS Scale Items	BAS-SLO Scale Items
Reward responsiveness subscale		
1	When I’m doing well at something, I love to keep at it.	When I’m doing well at something my partner values, I love to keep at it
2	When I get something I want, I feel excited and energized	When my partner tells me they love me, I feel excited and energized
3	When I see an opportunity for something I like, I get excited right away	When I see an opportunity to spend time with my partner, I get excited right away
4	When good things happen to me, it affects me strongly	When good things happen to my partner, it affects me strongly
5	It would excite me to win a contest	It would excite me for my partner and me to win a contest
Drive subscale		
1	I go out of my way to get things I want	I go out of my way to maintain my relationship with my partner
2	When I want something, I usually go all-out to get it	When it comes to maintaining my relationship with my partner, I usually go all-out
3	If I see a chance to get something I want, I move on it right away	If I see a chance to strengthen my relationship with my partner, I move on it straight away
4	When I go after something, I use a “no holds barred” approach	When it comes to maintaining my relationship with my partner, I use a “no holds barred” approach
Fun-seeking subscale		
1	I’m always willing to try something new if I think it will be fun	I’m always willing to try new things with my partner if I think it will be fun
2	I will often do things for no other reason than that they might be fun	I will often do things with my partner for no other reason than they are fun
3	I often act on the spur of the moment	I often act on the spur of the moment with my partner
4	I crave excitement and new sensations	I crave excitement and new sensations with my partner

**Table 2:** Means, standard deviations, skewness statistics, and kurtosis statistics for BAS-SLO Scale items in Study 1.

	M	SD	Skewness	Kurtosis
R1	3.58	0.56	−0.92	0.08
R2	3.67	0.55	−1.59	2.21
R3	3.56	0.62	−1.23	0.94
R4	3.57	0.64	−1.42	1.86
R5	3.56	0.66	−1.37	1.29
D1	3.21	0.77	−0.80	0.30
D2	3.12	0.76	−0.65	0.24
D3	3.37	0.71	−0.98	0.74
D4	2.84	0.78	−0.23	−0.039
F1	3.62	0.57	−1.33	1.55
F2	3.42	0.70	−1.00	0.44
F3	3.06	0.73	−0.36	−0.34
F4	3.34	0.69	−0.67	−0.22

R = Reward responsiveness subscale; D = Drive subscale; F = Fun-seeking subscale.

**Table 3:** Correlations among each BAS-SLO Scale subscale and the PLS-30 in Study 1.

BAS-SLO Scale Subscales	PLS-30
Reward	0.560 ***
Drive	0.418 ***
Fun-seeking	0.358 ***

*n* = 1554; *** *p* < 0.001.

**Table 4:** Correlations among variables used in Study 2 and their descriptive statistics.

Variable		1	2	3	4	5	6	7	8
1	Reward responsiveness	1	0.413 ***	0.465 ***	0.520 ***	−0.106 **	0.071 *	0.207 ***	0.450 ***
2	Drive		1	0.327 ***	0.468 ***	−0.048	0.099 **	0.308 ***	0.344 ***
3	Fun-seeking			1	0.340 ***	−0.087 *	0.035	0.121 ***	0.262 ***
4	PLS-30				1	−0.112 **	0.137 ***	0.465 ***	0.612 ***
5	Sex (male)					1	−0.064	−0.209 ***	−0.074 *
6	Months in love						1	0.063	0.232 ***
7	Obsessive thinking							1	0.330 ***
8	Commitment								1
	n					423			
	%					52.09			
	*M*	14.33	12.49	10.43	209.64		8.13	49.15	36.55
	*SD*	1.67	2.34	1.42	31.27		5.97	22.58	6.85

* *p* < 0.05; ** *p* < 0.01; *** *p* < 0.001.

**Table 5:** Hierarchical regression model of intensity of romantic love.

									95% *CI*	
	*R* ^2^	Adjusted *R*^2^	Adjusted *R*^2^ Change	*b*	*SE*	β	*t*	*p*	Lower	Upper
Step 1	0.453 ***	0.450 ***								
Sex				−0.772	1.668	−0.012	−0.463	0.644	−4.046	2.502
Months in love				−0.013	0.140	−0.002	−0.090	0.928	−0.288	0.263
Obsessive thinking				0.405	0.039	0.292	10.392	<0.001	0.328	0.481
Commitment				2.353	0.129	0.516	18.212	<0.001	2.099	2.607
Step 2	0.543 ***	0.539 ***	0.089 ***							
Sex				0.152	1.534	0.002	0.099	0.921	−2.860	3.164
Months in love				0.019	0.129	0.004	0.147	0.883	−0.234	0.272
Obsessive thinking				0.339	0.037	0.245	9.252	<0.001	0.267	0.410
Commitment				1.667	0.131	0.365	12.733	<0.001	1.410	1.924
Reward responsiveness				3.898	0.561	0.209	6.951	<0.001	2.797	4.999
Drive				2.131	0.370	0.159	5.753	<0.001	1.404	2.858
Fun-seeking				1.438	0.601	0.065	2.391	0.017	0.257	2.618

*n* = 812; *** *p* < 0.001.

**Figure A1:**
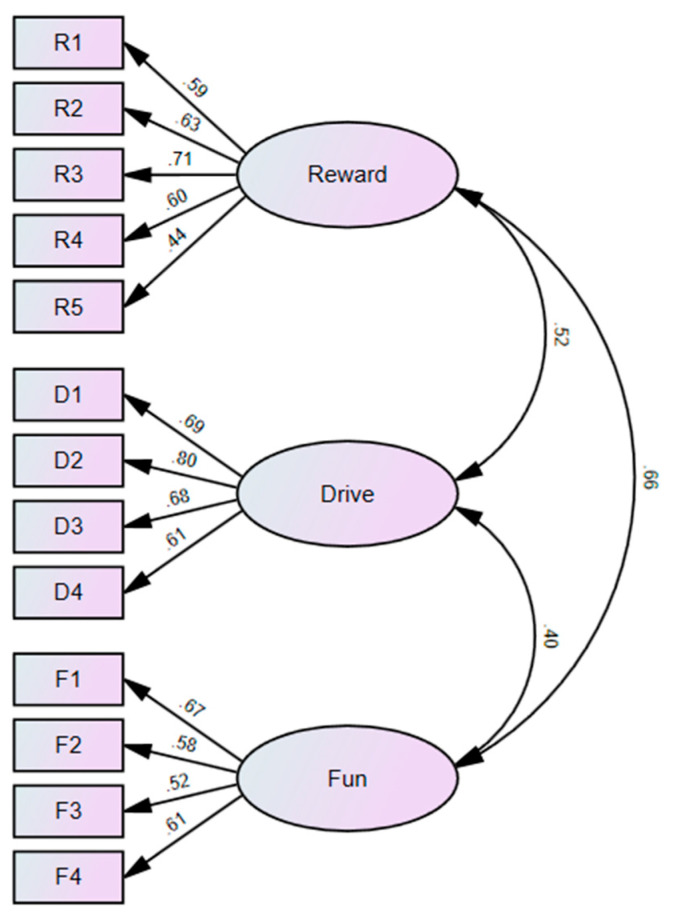
Results of the three-factor, 13-item CFA model for the BAS-SLO Scale. Note: Survey items (for description, see Table 3) are represented by rectangles, and latent factors are represented by ovals. Reward = reward responsiveness; Drive = drive; Fun = fun-seeking. The numbers above the one-directional arrows connecting factors to items represent standardized factor loadings. The numbers to the right of the bi-directional arrows connecting factors represent correlations between the factors.

## Data Availability

The data and code used this study are available form authors upon request.

## Appendix A

**Table A1:** Study 1 sample characteristics.

	Range	Mean	SD	Median	n	%
Sample					1556	100.00
Female					814	52.31
Heterosexual					1142	73.39
Age	18.00, 25.00	22.44	1.83	23.00		
PLS-30 (mean item; 1–9)	1.93, 9.00	7.02	1.13	7.17		
TLS-Commitment (mean item; 1–9)	1.80, 9.00	7.57	1.34	7.80		
Time thinking (%)	1.00, 100.00	49.68	23.45	50.00		
Dating, not co-habiting					479	30.78
Committed, not co-habiting					715	45.95
Committed, co-habiting					314	20.18
Married or de facto					48	3.08
Years in love (0–10 or more)	0.00, 10.00	1.90	2.15	1.00		
Relationship duration (years)	0.00, 10.00	1.82	2.05	1.00		
Relationship satisfaction (1–5)	1.00, 5.00	4.25	0.78	4.00		
HCL-32 (0–32)	0.00, 28.00	16.38	4.91	17.00		
Having sex (no/yes)					1377	88.62
Sex times per week	0.00, 30.00	3.03	2.79	2.00		
>13 years of education					1352	86.89
Current student (no/yes)					1046	67.22
Not working					629	40.42
Less than full-time work					491	31.56
Full-time work					436	28.02
AQOL-4D (12–48)	12.00, 35.00	17.59	3.48	17.00		

PLS-30 = Passionate Love Scale (divided by number of items); TLS-Commitment = five items from the TLS-15 Commitment subscale [55] but using a nine-point response option (divided by number of items); Obsessive thinking = Percent of time thinking about loved one; HCL-32 = Amended Hypomanic Checklist—32; AQOL-4D = Assessment of Quality of Life—4D; A small number of data points are missing among these variables.

**Table A2:** Country of residence of Study 1 participants.

Country	n	%
South Africa	182	11.70
Poland	136	8.74
Mexico	115	7.39
Portugal	111	7.13
Italy	110	7.07
UK and NI	109	7.01
Greece	93	5.98
USA	91	5.85
Spain	90	5.78
Germany	76	4.88
Hungary	67	4.31
Netherlands	67	4.31
Canada	43	2.76
Chile	36	2.31
France	31	1.99
Czech Republic	23	1.48
Slovenia	23	1.48
Australia	20	1.29
Belgium	20	1.29
Estonia	18	1.16
Latvia	18	1.16
Rest of sample	77	4.95

Rest of sample = Finland, Sweden, Austria, Ireland, Israel, New Zealand, Switzerland, Denmark, Japan, Norway, Luxembourg, South Korea.

## Appendix B

**Table A3:** Fit indices for one-factor and three-factor models of the BAS-SLO Scale in a sample of 1556 young adults self-reporting romantic love.

	CFI	RMSEA	SRMR
Criteria	>0.950 (0.900)	<0.060	<0.080
One-factor model	0.709	0.121	0.086
Three-factor model (13 items)	0.944	0.055	0.041
Three-factor model (11 items)	0.966	0.048	0.037

## Appendix D

**Table A4:** Final items of the proposed BAS-SLO Scale.

Item	BAS Scale Items	BAS-SLO Scale Items
**Reward responsiveness subscale**		
1	When I’m doing well at something, I love to keep at it.	When I’m doing well at something my partner values, I love to keep at it
2	When I get something I want, I feel excited and energized	When my partner tells me they love me, I feel excited and energized
3	When I see an opportunity for something I like, I get excited right away	When I see an opportunity to spend time with my partner, I get excited right away
4	When good things happen to me, it affects me strongly	When good things happen to my partner, it affects me strongly
**Drive subscale**		
1	I go out of my way to get things I want	I go out of my way to maintain my relationship with my partner
2	When I want something, I usually go all-out to get it	When it comes to maintaining my relationship with my partner, I usually go all-out
3	If I see a chance to get something I want, I move on it right away	If I see a chance to strengthen my relationship with my partner, I move on it straight away
4	When I go after something, I use a “no holds barred” approach	When it comes to maintaining my relationship with my partner, I use a “no holds barred” approach
**Fun-seeking subscale**		
1	I’m always willing to try something new if I think it will be fun	I’m always willing to try new things with my partner if I think it will be fun
2	I will often do things for no other reason than that they might be fun	I will often do things with my partner for no other reason than they are fun
3	I crave excitement and new sensations	I crave excitement and new sensations with my partner

## Appendix E

**Table A5:** Study 2 sample characteristics.

	Range	Mean	SD	Median	n	%
Sample					812	100.00
Female					389	47.91
Heterosexual					592	72.91
Age	18.00, 25.00	22.16	1.86	22.00		
PLS-30 (mean item; 1–9)	4.37, 9.00	6.99	1.04	7.10		
TLS-Commitment (mean item; 1–9)	2.00, 9.00	1.22	1.37	7.60		
Time thinking (%)	2.00, 100.00	49.10	22.58	49.00		
Dating, not co-habiting					358	44.09
Committed, not co-habiting					373	45.94
Committed, co-habiting					75	9.24
Married or de facto					6	0.74
Months in love (0–23)						
Relationship satisfaction (1–5)	1.00, 5.00	4.18	0.78	4.00		
HCL-32 (0–32)	0.00, 28.00	16.35	4.79	17.00		
Having sex (no/yes)					722	89.04
Sex times per week	0.00, 20.00	3.21	2.87	3.00		
>13 years of education					693	85.34
Current student (no/yes)					569	70.07
Not working					355	43.72
Less than full-time work					275	33.87
Full-time work					182	22.41
AQOL-4D (12–48)	12.00, 35.00	17.55	3.47	17.00		

PLS-30 = Passionate Love Scale (divided by number of items); TLS-Commitment = five items from the TLS-15 Commitment subscale [55] but using a nine-point response option (divided by number of items); Obsessive thinking = Percent of time thinking about loved one; HCL-32 = Amended Hypomanic Checklist—32; AQOL-4D = Assessment of Quality of Life—4D; A small number of data points are missing among these variables.

**Table A6:** Country of residence of Study 2 participants.

Country	n	%
South Africa	101	12.44
Poland	73	8.99
UK and NI	59	7.27
Portugal	57	7.02
Mexico	56	6.90
Greece	51	6.28
Germany	50	6.16
Spain	49	6.03
Italy	47	5.79
USA	39	4.80
Hungary	38	4.68
The Netherlands	31	3.82
Canada	22	2.71
Chile	22	2.71
France	16	1.97
Slovenia	14	1.72
Estonia	11	1.35
Australia	10	1.23
Czech Republic	10	1.23
Latvia	9	1.11
Austria	8	0.99
Rest of sample	39	4.80

Rest of sample = Finland, Ireland, Israel, New Zealand, Belgium, Sweden, Switzerland, Japan, South Korea, Denmark, Luxembourg, Norway.
